# Postpartum Depression and Risk Factors among Vietnamese Women

**DOI:** 10.1155/2018/4028913

**Published:** 2018-09-18

**Authors:** Thi Kim Ly Do, Thi Thanh Huong Nguyen, Thi Thu Huong Pham

**Affiliations:** ^1^Advanced Nursing Program, Hanoi Medical University, Vietnam; ^2^Faculty of Nursing and Midwifery, Hanoi Medical University, Vietnam

## Abstract

Postpartum depression (PPD) places a burden on maternal health. PPD exerts a negative impact on mothers' health and children's life. The purpose of this research was to identify the prevalence of PPD and the risk factors contributing to PPD. Therefore, a cross-sectional quantitative study was conducted. 116 women were categorized into two groups. One category included new mothers who received scores of Edinburgh Postpartum Depression Scale (EPDS) 12 or more. The other category included mothers who received scores less than 12. Descriptive statistic and then binary logistic regression were also performed. For EPDS ≥ 12, the prevalence of PPD was 27.6% among new mothers during the first year after delivery. Level of education, diseases during pregnancy, being the first-time mothers, dissatisfaction about family, and limited communication and interaction with others were significant predictors of PPD.

## 1. Background

Postpartum depression (PPD) is a major maternal health problem in the first year after giving birth. According to a study published in the British Journal of Psychiatry in 2017, the prevalence of PPD was 13-40% [1]. A study conducted in Vietnam indicated that the prevalence of PPD among new mothers was 20.4% in urban areas and was 15.8% in rural areas [2]. Previous studies have suggested that the mother's socioeconomic status and living the different areas create different prevalence of PPD [3]. Mothers living in poverty are more likely to be depressed and have greater barriers to access to treatment than the general population [4]. A research was conducted in Vietnam, indicated that 58% that seriously ill low-income patients who face higher health care costs would quit their treatment [5]. Depression is treatable but costly, so influencing quality of life of people [6]. Particularly in Vietnam healthcare system, the probability of satisfaction conditional on insurance reimbursement is lower for patients with residency status and higher for those without [7]. Nontreatment or nondiagnosis of PPD can have an adverse long-term effect [8]. Postpartum depression is associated with reducing the physical and mental health of women's lives [9]. PPD may also lead to lower cognitive and linguistic development in the first year of life for the child(ren) [10], and they might experience behavioral disorder and impair physical development [11, 12]. Suicide is known to be one of the worst consequences of PPD [13, 14]. Psychoses occur in 1 to 2 per 1000 postpartum women; they may present as schizophrenic or affective disorder or as confusional states [15, 16]. Numerous studies have demonstrated many factors associated with PPD including obstetric history, mode of delivery [17], biochemical genetic [18, 19], and other social stressors: age [20]; socioeconomy [21, 22]; culture [23]; education; negative life events. To prevent, early treatment of PPD is likely to reduce public sector costs, increase earnings, and improve quality of life for women [24].

## 2. Method

This was a cross-sectional study conducted from August 20 to September 7 in Hanoi. This study will include all women whose child(ren) are less 1 year old and they are over 18 years old. 116 postpartum women were screened for postpartum depression using Edinburgh Postpartum Depression Scale (EPDS). The EPDS includes 10 items measured in a Likert scale of 0-3. Score of 12 and above indicates risk factors of depression. The sensitivity and specificity of EPDS were 65-100% and 49-100%, respectively [25]. EPDS has been translated and validated for non-English speaking population [26, 27] and has used widely in Asian [28, 29]. New mothers were also asked to report information about demographics, pregnancy and disease history, interpersonal skill, and characteristics.

Prior to the main survey, 10 new mothers were asked for a pilot interview to check for readability and clarity of the questionnaire. The face-to-face interview was conducted at participant's private house.

SPSS software version 23.0 was employed for data management and analysis. Descriptive statistic and then binary logistic regression were also performed.

The study was approved by ethics committee of Hanoi Medical University, Decision No. 5042 on November 7, 2017.

## 3. Results

A score of EPDS cut-off was ≥ 12 and the prevalence of postpartum depression among 116 new mothers (18-43 years old) during the first year after childbirth was 27.6 % (Table 1).

The majority of women were over 25 years old and graduated from college or higher (N = 116, 59.5%). There are 66 women who lived in rural areas (56.9%) and 50 women who lived in urban areas (43.1%). In this study, most people are nonreligious (88.8%) and just 11.2% have religions such as Buddhism, Christianty, and others.

1. Chronic diseases including diabetes, cardiovascular diseases, hypotension, hypertension, anemia, Parkinson, Lupus immunity disorder, and cancer.
2. Diseases during pregnancy including diabetes, hypertension/hypotension, hepatitis, chicken pox, and gynecological inflammation.
3. Life-event including family member died or critical diseases, accident, and unfavorable condition.

An increased risk of maternal depressive symptoms was remarkably associated with the level of education (OR 0.4, p = 0.036). The odd ratio for this variable, however, is 0.4, a value less than 1. This indicates that new mothers with a lower level of education were less likely to experience PPD. The diseases during pregnancy contracted by the mother are also a significant predictor, according to sig. value (p = 0.009). In the logistic regression model, the odd ratio of new mothers without gynecological diseases during pregnancy was 0.3 times less than likely to those experiencing diseases during pregnancy. Another variable in Table 2 found to be associated with the PPD's symptoms was whether they were first-time mothers. This indicated that those who became first-time mothers were over 2 times more likely to suffer from PPD than those who already had children.

Our results found that the variables for increasing EPDs were the satisfaction of new mothers about family life (p < 0.001). Postpartum women who were feeling happy were 0.2 times less likely to present with symptoms of PPD than the mother having arguments in their family. New mothers with limited communication and interaction with others were over 4 times more than the reference categories (p = 0.001, OR 4.4).

## 4. Discussion

Postpartum depression (PPD) is a major maternal health problem in the first year after giving birth. The prevalence of postpartum depression was 27.6% as measured by the Edinburgh Postpartum Depression Scale (EPDS) with a cut-off point ≥ 12. Our study found the incidence of PPD was higher than it was estimated in Thua Thien Hue Province (18.1%) [2] and Da Nang (19.3%) [30]. Many studies have reported PPD rates from 18 countries [31]; the average lifetime and 12-month prevalence estimates of major depression were 14.6% and 5.5% in high-income countries and 11.1% and 5.9% in low- to middle-income countries. The result of our study did not correspond to some researches about the relationship between PPD and regional differences (rural areas and urban areas). These differences may be related to the mothers' characteristics (demographic, pregnant history, and interpersonal characteristics) and study design (sample, method, and cut-off point).

In our study, women with gestational diseases are at higher risk factor of PPD compared to women who have not. The causes of the influence of diseases (gestational diabetes, gynecological inflammation, hypertension or hypotension, and hepatitis) on increasing PPD are not clear [32, 33]. It is possible that the diseases in pregnancy seem to be a psychological burden for women, with remarkable effects on developing PPD. For instance, one of some studies reviewed of the literature regarding gestational diabetes and PPD suggests that the percentage of women reporting PPD following a diagnosis of gestational diabetes was higher than women who did not [34].

Two strong predictors of developing PPD in this study were limited communication/interaction with others when struggling (p = 0.001) and dissatisfaction about their family life (p < 0.001). Happiness and satisfaction with their life may prevent the development of PPD. Some theories explained the mechanism of emotional associated with PPD's development, “including the monaamine, biorhym, neuroendocrine, neuroimmune, and kindling/neuroplasticity theories” [35]. Many researchers hold the belief that the hormones (endorphin, serotonin, and dopamine) are released when having positive thinking and emotion [18, 36]. Women who communicate and interact with others during the postpartum period are at a lower risk of increasing PPD. The previous studies described relationship between stress and limited relationship, as well as new mothers who do not want to communicate and share something with others [8, 37]. The results from many researchers are similar to our findings.

However, the study was conducted in a short time (from August 20 to September 7, 2017) and a small sample size (N = 116). Stigma, perceptions of motherhood, religion, and culture sensitively were major barriers to screening for PPD.

## 5. Conclusion

This study makes important contributions to knowledge about the prevalence of PPD in Hanoi and the risk factors associated with PPD. The prevalence of PPD during the first year after childbirth was 27.6 %. PPD is considered as a burden in the community. Several therapies are recommended to reduce the prevalence of PPD: conventional face-to-face therapy [38] (the range of pharmacological, psychotherapeutic, and other nonpharmacologic interventions); a possibility of mobile based therary [39].

## Figures and Tables

**Table 1:** Demographic.

**Variable**		**Number (N=116)**	**Percentage (%)**
**Score of EPDS**	≥12	32	27.6
	< 12	84	72.4

**Age**	≤ 24	30	25.9
	> 24	86	74.1

**Education**	High school graduate and lower	47	40.5
	College and higher	69	59.5

**Address**	Rural	66	56.9
	Urban	50	43.1

**Religion**	Yes	13	11.2
	No	103	88.8

**Table 2:** Factors associated with PPD.

**Variables **		**EPDS**		**95% CI**	**OR**	**p-value**
		**≥12**	**<12**			
**Demographic factors**						

**Age**	≤ 24	8	22	0.4-2.4	0.9	0.89
	> 24	24	62		Refer	
**Education**	High school graduate and lower	8	39	0.2-0.9	0.4	**0.04**
	College and higher	24	45		Refer	
**Address**	Rural	16	50	0.3-1.5	0.7	0.35
	Urban	16	34		Refer	
**Religion**	Yes	2	11	0.5-10.8	Refer	0.29
	No	30	73		2.3	

**Pregnant history**						

**Chronic disease (1)**	Yes	7	12	0.2-1.7	Refer	0.32
	No	25	72		0.6	
**Gynecological diseases during pregnancy (2)**	Yes	11	11	0.1-0.8	Refer	**0.01**
	No	21	73		0.3	
**First-time mother**	Yes	22	39	1.1-6.0	2.5	**0.03**
	No	10	45		Refer	
**Breastfeeding**	Yes	31	80	0.2-14.4	1.6	0.69
	No	1	4		Refer	
**Planned pregnancy**	Planned	25	76	0.1-1.1	0.4	0.07
	Unplanned	7	8		Refer	

**Interpersonal characteristics**						

**Family structure**	Extended	21	47	0.6-3.5	1.5	0.34
	Nuclear	11	37		Refer	
**Satisfaction about family life**	Feeling happy	8	55	0.1-0.4	0.2	**0.000**
	Having arguments	24	29		Refer	
**Communicate and interact with other**	Limited than before	23	31	1.8-10.6	4.4	**0.001**
	As before	9	53		Refer	
**Tell with others when struggling**	Yes	5	4	0.9-14.8	3.7	0.05
	No	27	80		Refer	
**Having stressful life-events (3)**	Yes	23	67	0.2-1.6	Refer	0.36
	No	9	17		0.6	

## Data Availability

The data used to support the findings of this study are available from the corresponding author upon request.

## Abbreviations

PPD: Postpartum depression
EPDS: Edinburgh Postpartum Depression Scale.
